# Pharmacological and Structure-Activity Relationship Evaluation of 4-aryl-1-Diphenylacetyl(thio)semicarbazides

**DOI:** 10.3390/molecules19044745

**Published:** 2014-04-16

**Authors:** Monika Wujec, Ewa Kędzierska, Edyta Kuśmierz, Tomasz Plech, Andrzej Wróbel, Agata Paneth, Jolanta Orzelska, Sylwia Fidecka, Piotr Paneth

**Affiliations:** 1 Department of Organic Chemistry, Medical University, Chodźki 4a, 20-093 Lublin, Poland; E-Mails: monika.wujec@umlub.pl (M.W.); edyta.kusmierz@umlub.pl (E.K.); tomasz.plech@umlub.pl (T.P.); agata.siwek@umlub.pl (A.P.); 2 Department of Pharmacology and Pharmacodynamics, Medical University, Chodźki 4a, 20-093 Lublin, Poland; E-Mails: ewa.kedzierska@umlub.pl (E.K.); jolanta.orzelska@umlub.pl (J.O.); sylwia.fidecka@umlub.pl (S.F.); 3 History of Medical Sciences Department, Medical University, Szkolna 18, 20-124 Lublin, Poland; E-Mail: wand@bg.umlub.pl; 4 Institute of Applied Radiation Chemistry, Technical University of Lodz, Zeromskiego 116, 90-924 Lodz, Poland; E-Mail: piotr.paneth@p.lodz.pl

**Keywords:** (thio)semicarbazides, conformational analysis, electrostatic properties, CNS activity, analgesic activity, serotonergic activity

## Abstract

This article describes the synthesis of six 4-aryl-(thio)semicarbazides (series **a** and **b**) linked with diphenylacetyl moiety along with their pharmacological evaluation on the central nervous system in mice and computational studies, including conformational analysis and electrostatic properties. All thiosemicarbazides (series **b**) were found to exhibit strong antinociceptive activity in the behavioural model. Among them, compound 1-diphenylacetyl-4-(4-methylphenyl)thiosemicarbazide **1b** was found to be the most potent analgesic agent, whose activity is connected with the opioid system. For compounds from series **a** significant anti-serotonergic effect, especially for compound 1-diphenylacetyl-4-(4-methoxyphenyl)semicarbazide **2b** was observed. The computational studies strongly support the obtained results.

## 1. Introduction

It is well known that many diseases are accompanied by inflammation and pain. Therefore, the search for new compounds with a large spectrum of biological activities is an important focus of attention for chemists as well as for pharmacologists. Semicarbazides and thiosemicarbazides appear to be ideal candidates for pharmacologically significant scaffolds. It follows from the literature that thiosemicarbazide derivatives possess a wide range of pharmacological activities such as antituberculosis [1,2], antiviral [3,4], anti-inflammatory [5], anticonvulsant [6,7,8], analgesic [5], antibacterial [9,10,11], antifungal [12,13], and anticancer properties [14].

Semicarbazides are also an important class of molecules with a large spectrum of biological properties. These compounds have been studied as anticonvulsant [15], antitubercular [16] and antinociceptive [17,18,19] agents. Additionally semicarbazides with aryl substitution are reported to display excellent anticonvulsant activity in mice and rats compared to that of phenytoin [20]. It is important to note that some of tested compounds showed lesser CNS depression and behavioural despair side effects than the conventional antiepileptic drugs [7], and were defined as lead molecules in the design of potent anticonvulsant drugs [8]. Based on these facts, many (thio)semicarbazides and their cyclic analogues have been synthesized in our laboratory and their antimicrobial and CNS system bioactivity was studied [10,21,22]. This work is continuation of our efforts to find bioactive (thio)semicarbazides with central nervous system pharmacological activity in mice.

## 2. Results and Discussion

### 2.1. Chemistry

**Scheme 1:**
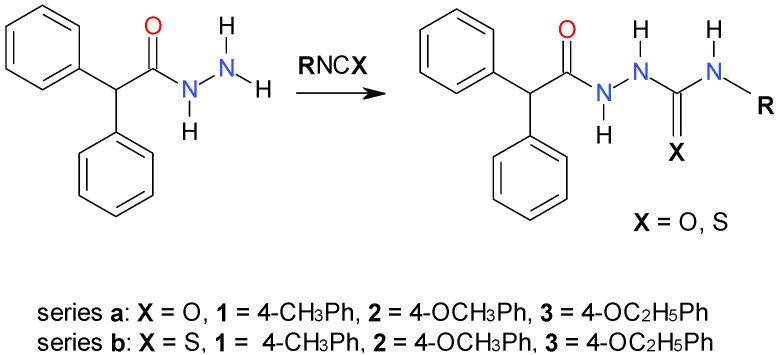
Synthetic route for 4-aryl-1-diphenylacetylsemicarbazides (series **a**) and 4-aryl-1-diphenylacetylthiosemicarbazides (series **b**).

The synthetic route employed for the preparation of the title 4-aryl-1-diphenylacetyl(thio)semicarbazides (series **a** and **b**) is shown in Scheme 1. As can be seen, a simple synthesis was carried out, starting from the commercially available diphenylacetic acid hydrazide and the appropriate iso(thio)cyanates. At the end of the reaction, the isolated (thio)semicarbazide derivatives were obtained as white coloured solids. This procedure was adapted from an procedures previously reported by Kusmierz *et al.* [23] and Wujec *et al.* [24] and gave us satisfactory yields.

### 2.2. Pharmacological Activity

The results of the pharmacological investigation showed that both groups of tested compounds—semicarbazides (series **a**) and thiosemicarbazides (series **b**)—exerted significant influence on the central nervous system (CNS) of laboratory animals. All substances exhibited very low toxicity: over 2,000 mg/kg (i.p.), therefore an ED_50_ = 2,000 mg/kg was accepted, and regressive doses of 200 and 100 mg/kg (i.p.; 0.1 and 0.05 ED_50_ respectively) were used for further studies.

**Figure 1:**
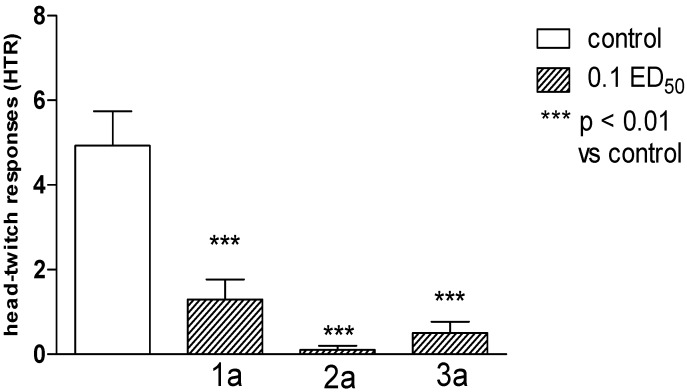
The influence of **1a**, **2a** and **3a** on “head-twitch” responses (HTR) evoked by l-5-HTP (200 mg/kg). One-way ANOVA showed significant changes in the number of HTR [F_(3.40)_ = 16.49; *p* < 0.0001]. Post hoc Dunnett’s test confirmed a significant reduction in HTR of mice after the administration of the compound **1a**, **2a** and **3a** in the dose of 0.1 ED_50_ (*p* < 0.001).

Semicarbazide derivatives (series **a**) seem to affect serotonergic neurotransmission, since all compounds inhibited significantly l-5-HTP-induced head-twitches (Figure 1). The drug-elicited head-twitch response (HTR) [25,26] is a selective behavioural model for 5-HT_2_ agonist activity in rodents, and several previous studies have established that direct and indirect 5-HT agonists induce this effect [27,28,29,30,31,32]. Furthermore, 5-HT_2_ receptor antagonists selectively block HTR [30,33,34], and their potency is highly correlated with the antagonist’s affinity for 5-HT_2_ receptors [25,35].

**Figure 2:**
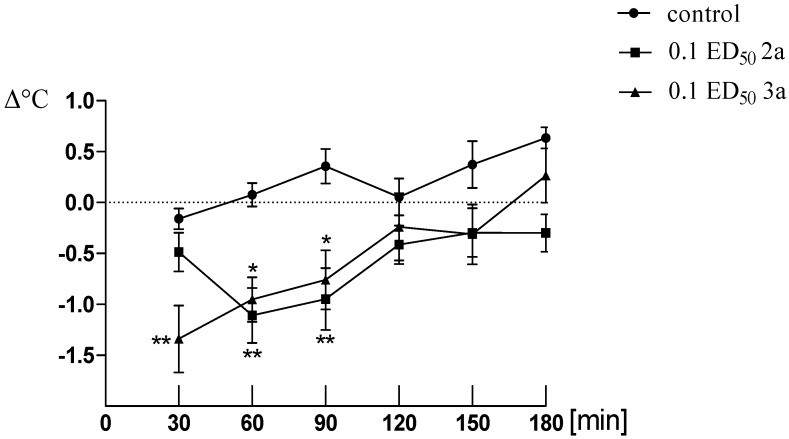
The influence of compound **2a** and **3a** (used in a dose of 0.1 ED_50_) on the body temperature of the mice. Two-way ANOVA revealed significant effects for both compounds [F_(2.142)_ = 22.14; *p* < 0.0001] and time [(F_(5.142)_ = 6.22; *p* < 0.0001], but not interaction [F_(10.142)_ = 1.57; *p* = 0.122]. Post hoc Bonferroni test confirmed a significant decrease in the body temperature of the mice after the administration of compound **2a** in the dose of 0.1 ED_50_ in 60 and 90 min (*p* < 0.001), and compound **3a** in the dose of 0.1 ED_50_ in 30 min (*p* < 0.001), 60 and 90 min (*p* < 0.05).

In addition, compounds **2a** and **3a** substantially decreased the body temperature of normothermic mice (Figure 2), which also may confirm the involvement of serotonergic system. Serotonin has been reported to play an important role in central regulation of body temperature [36]. The MAO (monoamine oxidase) type A inhibitors appear to be crucially involved in hypothermia [37]. Hypothermia in rodents has been reported for MAO (monoamine oxidase) type A enzyme inhibitors such as clorgyline [38] and harman (1-methyl-β-carboline) [39]. As a result of MAO-inhibition, 5-HT levels in the body are increased and may precipitate a serotonin syndrome.

**Figure 3:**
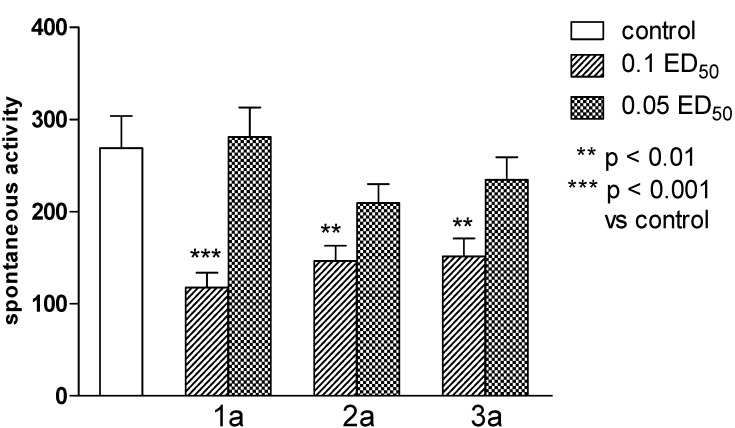
The influence of **1a**, **2a** and **3a** on the spontaneous locomotor activity of mice. One-way ANOVA showed significant changes in locomotor activity of mice [F_(6.49)_ = 6.857; *p* < 0.0001]. The post hoc Dunnett’s test confirmed a significant reduction in motility of mice after the administration of the compound **1a** in the dose of 0.1 ED_50_ (*p* < 0.001) and compounds **2a**, **3a** in the dose of 0.1 ED_50_ (*p* < 0.01).

All series **a** compounds decreased the locomotor activity of the mice (Figure 3), which seem to suggest their depressive effects on the CNS [40], while none of them changed the hyperactivity caused by administration of amphetamine. Tested series **a** compounds did not show antinociceptive activity in a “writhing” test.

**Figure 4:**
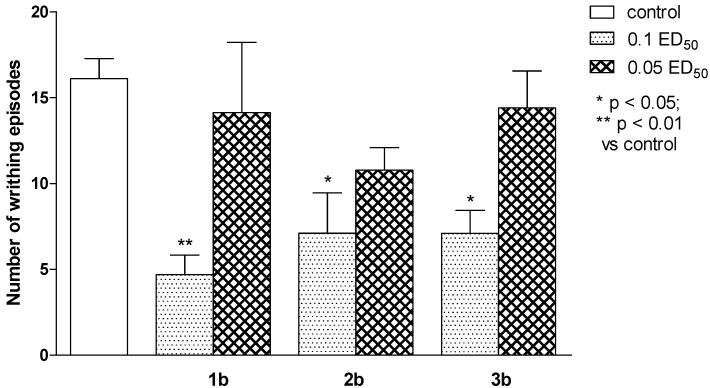
The antinociceptive effects of **1b**, **2b** and **3b** assessed in the ‘writhing’ test in mice. One-way ANOVA showed significant changes in the number of writhing episodes of mice after the administration of compounds 1b, 2b and 3b (F_6.58_ = 4.725, *p* < 0.001). Post hoc Dunnett’s test confirmed a significant reduction in the writhing episodes of mice after the administration of the compound **1b** in the dose of 0.1 ED_50_ (*p* < 0.01) and compounds **2b** and **3b** in the dose of 0.1 ED_50_ (*p* < 0.05).

**Figure 5:**
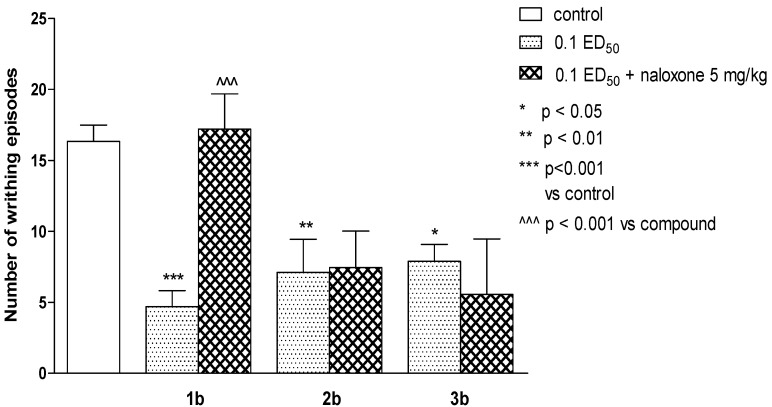
The influence of naloxone, 5 mg/kg, s.c. on antinociceptive activity of **1b**, **2b** and **3b** evaluated in the “writhing” test. One-way ANOVA showed significant changes in the number of writhing episodes of mice after the administration of compounds 1b, 2b and 3b (F_6.67_ = 6.589, *p* < 0.0001). *Post hoc* Dunnett’s test confirmed a significant reduction in the writhing episodes of mice after the administration of the compound **1b** in the dose of 0.1 ED_50_ (*p* < 0.001), **2b** in a dose of 0.1 ED_50_ (*p* < 0.01) and **3b** in a dose of 0.1 ED_50 _(*p* < 0.05). Naloxone inhibited antinociceptive action of **1b** (*p* < 0.001 *vs.* compound **1b**; post hoc Dunnett’s test).

Thiosemicarbazide derivatives (series **b**) exerted antinociceptive activity on mice in the “writhing” test, with compound **1b** (in the dose of 0.1 ED_50_) causing the strongest reaction (Figure 4). Naloxone completely reversed this effect which seems to suggest the endogenous opioid system’s contribution (Figure 5) [41]. In other performed tests, these substances were not active.

Both investigated series of compounds **a** and **b** did not cause any coordination impairments as measured in the rota-rod and chimney tests. These results show the lack of neurotoxicity, and muscle relaxant potency of the substances, this is important because it can change the results of other tests (e.g., motility test) and affecting reliability of the tests results [38]. Moreover, pentetrazole-induced seizures were not affected by the compounds of either series.

### 2.3. Computational Part

It is not surprising that high protein(s) specificity results in significantly different biological activity of closely related compounds. Nonetheless frequently computational modeling allows to rationalize observed bioactivities and provides background for rational design of molecules with expected bioactivity. In particular, electrostatic properties have been recently correlated with the binding affinity of low molecular weight heterocycles to various receptors and enzymes involved in CNS. In the light of these facts, we have compared the electrostatic and geometrical properties of studied compounds in hope of gaining some insight into their bioactivity.

**Figure 6:**
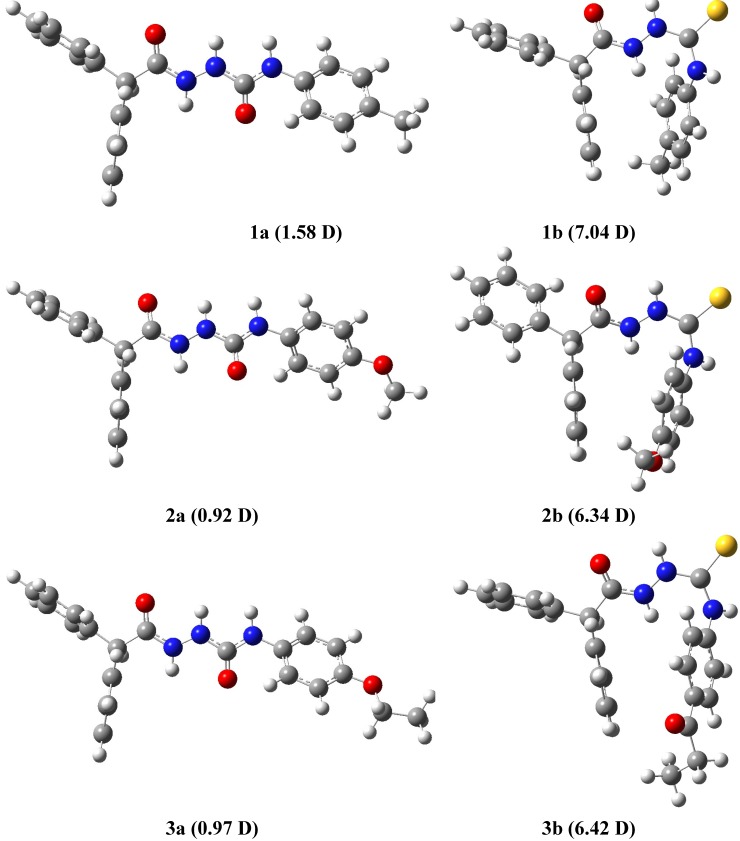
Molecular shapes of semicarbazides **a** (**left**) and thiosemicarbazides **b** (**right**) optimized at the molecular mechanics level with OPLS force field.

**Figure 7:**
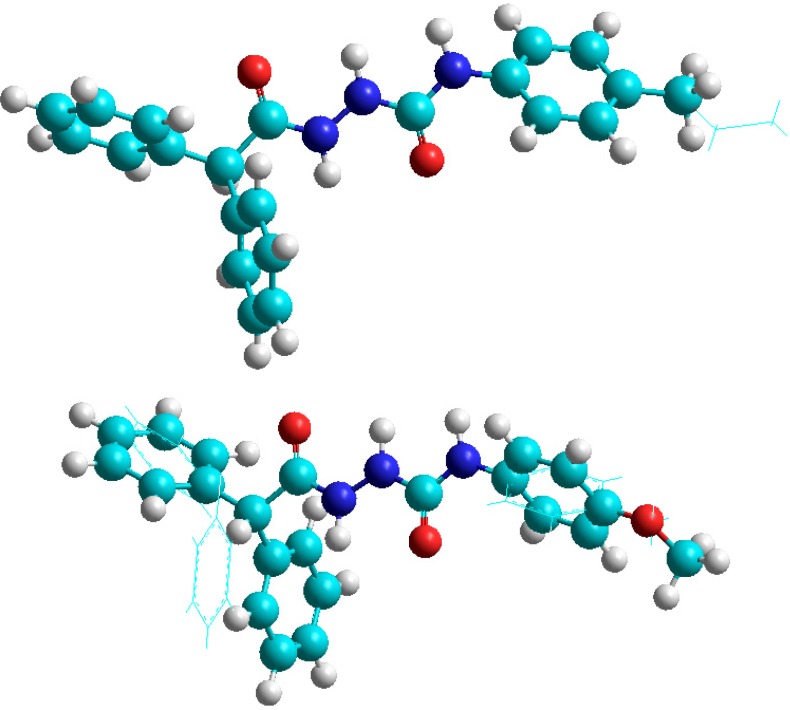
Overlay of structures of **1a** (balls and sticks) and **3a** (sticks) (**top**) and **1a** (sticks) and **2a** (balls and sticks) (**bottom**).

The structures of molecules from series **a** and **b** were optimized at the molecular mechanics level with the OPLS force field which has been shown to correctly described both the geometries and the energetic of these classes of compounds [42]. As illustrated in Figure 6, OPLS-optimized structures of semicarbazides **a** and thiosemicarbazides **b** exhibit some differences: both oxygen atoms of the C(=O)–NH–NH–C–(=O) core in **a** are located on opposite sides of molecule (a “*trans*”conformation) while the oxygen and sulphur atoms of the C(=O)–NH–NH–C–(=S) core in **b** are on the same sides (“*cis*” conformation). Another observation that comes from the analysis of the presented structures is that the benzoyl ring at N4 position of semicarbazide **a** is almost in the same plane with the C(=O)–NH–NH–C–(=O) core, while in **b** it is nearly perpendicular to C(=O)–NH–NH–C–(=S) (Figure 1). These results together might imply that differentiation of the biological activity of tested semicarbazides **a** and thiosemicarbazides **b** originates in the molecular shape. Following this finding, we analyzed the geometry of **a** and **b** in details. As shown in Figure 7, the geometries of **1a** and **3a** (Figure 7, top) are practically identical, but they differ significantly from the most active anticonvulsant agent **2a**, mainly in the orientation of phenyl rings (Figure 7, bottom). Thus, the geometry of molecule might be important for the antiserotonergic activity of semicarbazide derivatives **a**. Unfortunately, comparison of the molecular shape of thiosemicarbazides **b** did not show any recognizable relationship with their antinociceptive activity (data not shown).

The assumption of the existence of the relationship between bioactivity of studied (thio)semicarbazides **a** and **b** and their molecular structure can be further inferred from the corresponding dipole moments of the molecules. As seen in Figure 6, dipole moments for series **a** are 1.58, 0.92, 0.97 D, while for series **b** are 7.04, 6.34, 6.42 D, respectively. These values show that electrostatic distribution in the series **a** differs significantly from that in the series **b**. This can be explained mainly by the geometry of series **a** that, as it was mentioned above, differs significantly from that in the series **b**.

**Figure 8:**
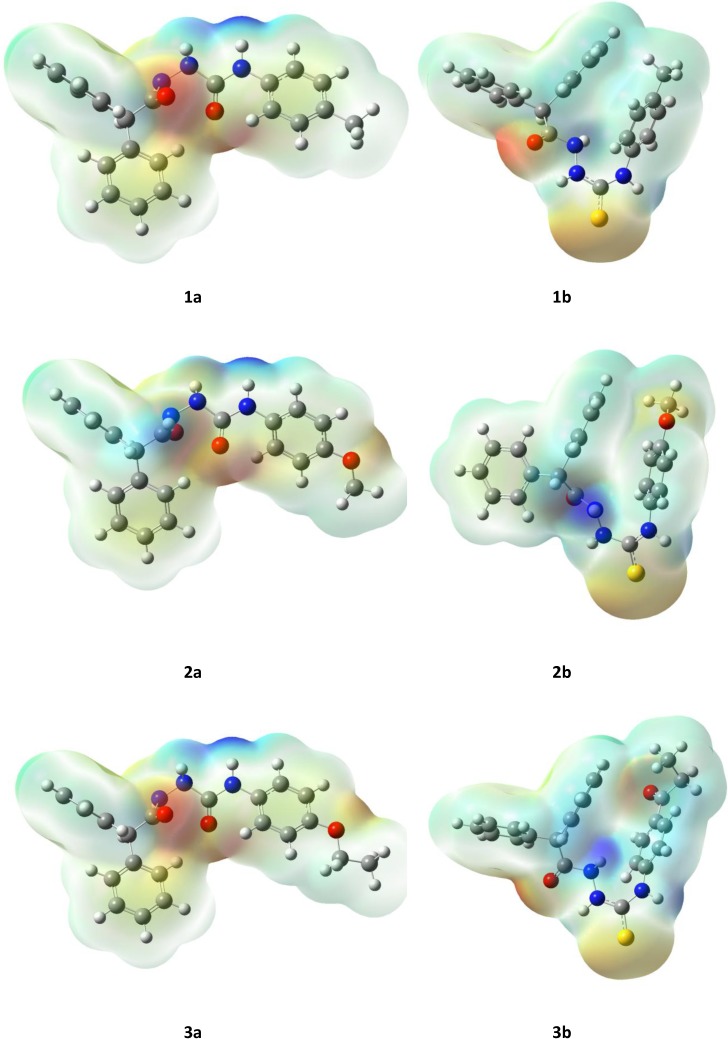
The electrostatic potential surfaces of semicarbazides **a** and thiosemicarbazides **b**.

Within series **a** the best antiserotonergic activity was noted for derivative with the lowest dipole moment value (compound **2a**). This suggest that the dipole moment might be a good descriptor for the (Q)SAR studies relating antiserotonergic activity to the structure. Unfortunately, no close correlation could be deducted from the generated electrostatic potential surfaces (Figure 8). When the antinociceptive activity of thiosemicarbazides **b** is considered, it seems that the observed bioactivity correlates with charge density around the oxygen atom of the methoxy (compound **2b**) and ethoxy group (compound **3b**). Its lack in **1b** may be responsible for its higher activity (Figure 8).

## 3. Experimental

### 3.1. General Information

The reagents were purchased from Sigma-Aldrich (St. Louis, MS, USA) or Lancaster (Ward Hill, NY, USA) and were used without further purification. The melting points were determined on a Fisher-Johns block and are uncorrected. Elemental analyses (within ±0.4% of theoretical values) were determined by a AMZ-CHX elemental analyzer (PG, Gdańsk, Poland). IR spectra was recorded in KBr using a Specord IR-75 spectrophotometer (Carl Zeiss, Jena, Germany). ¹H-NMR spectrum was recorded on a Bruker Avance (300 MHz) spectrometer (Bruker BioSpin GmbH, Rheinstetten, Germany). Analytical thin layer chromatography was performed with Merck 60F_254_ silica gel plates (Merck Co., Darmstadt, Germany) visualized by UV irradiation (254 nm).

### 3.2. Chemistry

#### 3.2.1. General Procedure for the Synthesis of 4-aryl-1-Diphenylacetylsemicarbazides **1a** and **2a**

A reaction mixture of diphenylacetic acid hydrazide (0.01 mol) and related isocyanate (0.01 mol) in diethyl ether (10 mL) was kept in room temperature for 24 h, then the compound formed was filtered off and crystallized from ethanol.

*1-Diphenylacetyl-4-(4-methylphenyl)semicarbazide* (**1a**). Yield: 93%. Mp. 205–207 °C. ^1^H-NMR (DMSO-*d_6_*) δ (ppm): 2.49 (s, 3H, CH_3_); 5.00 (s, 1H, CH); 6.95–7.36 (m, 14H, CH_arom_); 8.10 (s, 1H, NH); 8.63 (s, 1H, NH); 10.15 (s, 1H, NH). IR (KBr, *ν*, cm^−1^): 3350, 2800, 1675, 1540, 1478, 1355. Anal. Calc. for C_22_H_21_N_3_O_2_ (359.42): C 73.52, H 5.89, N 11.69. Found: C 73.48, H 5.77, N 11.70.

*1-Diphenylacetyl-4-(4-methoxyphenyl)semicarbazide* (**2a**). Yield: 89%. Mp. 194–196 °C. ^1^H-NMR (DMSO-*d_6_*) δ (ppm): 3.32 (s, 3H, OCH_3_); 5.00 (s, 1H, CH); 6.82–7.35 (m, 14H, CH_arom_); 8.04 (s, 1H, NH); 8.53 (s, 1H, NH); 10.12 (s, 1H, NH). IR (KBr, *ν*, cm^−1^): 3356, 2800, 1680, 1540, 1488, 1360. Anal. Calc. for C_22_H_21_N_3_O_3_ (375.42): C 70.38, H 5.64, N 11.19. Found: C 70.45, H 5.67, N 11.25.

#### 3.2.2. Procedure for the Synthesis of 4-(4-Ethoxyphenyl)-1-(diphenylacetyl)semicarbazide **3a** and 4-Aryl-1-(diphenylacetyl)thiosemicarbazides **1b–3b**

A reaction mixture of diphenylacetic acid hydrazide (0.01 mol) and 4-ethoxyphenylisocyanate or related isothiocyanate (0.01 mol) was heated in an oil bath at 70–80 °C and progress of reaction was monitored by thin layer chromatography. After 10–12 h, the reaction was completed and the crude reaction mixture was washed with diethyl ether and crystallized from ethanol. Physicochemical data for compounds **1b**, **2b**, and **3b** were presented in our previous papers [21].

*4-(4-Ethoxyphenyl)-1-diphenylacetylsemicarbazide* (**3a**). Yield: 93%. Mp. 205–207 °C. ^1^H-NMR (DMSO-*d_6_*) δ (ppm): 1.27, 1.29, 1.31 (t, 3H, CH_3_, *J* = 6.9 Hz); 3.92, 3.94, 3.96, 4.01 (q, 2H, CH_2_, *J* = 6.9 Hz); 5.00 (s, 1H, CH); 6.81–7.34 (m, 14H, CH_arom_); 8.05 (s, 1H, NH); 8.53 (s, 1H, NH); 10.14 (s, 1H, NH). IR (KBr, *ν*, cm^−1^): 3365, 2805, 1680, 1530, 1460, 1340. Anal. Calc. for C_23_H_23_N_3_O_3_ (389.45): C 70.93, H 5.95, N 10.79. Found: C 70.98, H 5.90, N 10.70.

### 3.3. Pharmacology

The experiments were performed on male Albino Swiss mice (16–30 g). 8–10 animals were kept in a cage, at room temperature of 22 ± 1 °C, on a natural dark-light cycle with free access to food (LSM, Motycz, Poland) and water. All experiments were performed in accordance with the opinion of The Local Ethics Committee for Animal Experimentation.

All substances were administered intraperitoneally (i.p.), as suspensions in aqueous solution of 0.5% methylcellulose (tylose) and were injected 60 min before tests. In the “writhing” procedure, the investigated compounds were injected subcutaneously (s.c.) because the acetic acid (0.6%) was administered i.p. All substances were given in a volume of 0.1 mL per 10 g of body weight. The control animals received an equivalent volume of the solvent at the respective time before the test. All tests performed, suggested by [38], are generally accepted as basic in investigation of the central activity by behavioural methods. The acute toxicity of the compounds were assessed acc. to Litchfield and Wilcoxon method [43], as the ED_50_ calculated as “the loss of righting reflex” within 48 h. The compounds were injected in doses equivalent to 0.1 ED_50_ (200 mg/kg) and 0.05 ED_50_ (100 mg/kg). In addition, the activity of the compounds was assessed in the following tests:

*Locomotor activity* was measured in photocell apparatus (round Plexiglas cage, 32 cm, Multiserv, Lublin, Poland) for a single mouse for 30 min as:

(a) spontaneous activity
(b) amphetamine-induced hyperactivity: mice received s.c. 5 mg/kg of amphetamine 30 min before the test;

*Nociceptive reactions* were studied in the acetic acid (0.6%)—Induced “writhing” test [44]—The number of writhing episodes was measured for 10 min, starting 5 min after i.p. administration of acid solution;

*Motor coordination* was evaluated in the rota-rod [45]—motor impairments, defined as the inability to remain on the rotating rod for 1 min were measured and the mean time spent on the rota-rod was counted for each mouse, and in the chimney test [46]—motor impairments were indicated by the inability to perform the test within 1 min;

*Body temperatue in normothermic mice* was measured in the rectum of animals with a thermistor thermometer and was recorded 30, 60, 90, 120, 150 and 180 min after the injection of investigated compounds in the doses of 0.1 and 0.05 ED_50_ i.p.;

*Pentylenetetrazole-induced* *convulsions* (PTZ, 110 mg/kg, s.c.) were evaluated as the number of mice with clonic seizures, tonic convulsions and dead animals;

*Head twitch responses (HTR) after 5-hydroxy-l-tryptophan (l-5-HTP)* *administration*, were estimated acc. to Corne *et al.* [23]. Mice received l-5-HTP (200 mg/kg, i.p.) and the number of HTR was recorded in 6 two-minutes intervals (4–6, 14–16, 24–26, 34–36, 44–46, 54–56 min) during 1 h;

*Influence of naloxone* *on the antinociceptive effect* (5 mg/kg, s.c.) of the compounds was assessed in the “writhing” test.

### 3.4. Statistics

Obtained data were calculated by Fisher exact test (PTZ-induced seizures), two-way analysis of variance (ANOVA) followed by Bonferroni *post hoc* test (body temperature) and one-way ANOVA followed by Dunnett’s *post hoc* test (other tests). All results are presented as means ± SEM. *p* < 0.05 was considered as statistically significant.

### 3.5. Computational Part

Computational procedure described previously [9] has been followed; extensive conformational searches were carried out using the molecular mechanics level with OPLS [47] force field as implemented in HyperChem8.0.3 [48]. The most stable structures obtained were subsequently optimized to the closest local minimum at the semiempirical level using RM1 parametrization. Convergence criteria were set to 0.1 and 0.01 kcal/mol/Å for OPLS and RM1 calculations, respectively. Electrostatic potentials were calculated and visualized using Gaussian 09 and GaussView 5 [49], respectively at the HF/6-31G level [50].

## 4. Conclusions

In this contribution, a pharmacological evaluation of the activity of six (thio)semicarbazide derivatives on the central nervous system in mice was performed. The results suggest a possible involvement of the serotonin system in the effects of compounds series **a**, whereas the opioid system apppears to be involved in the effects of compound **1b**. The precise mechanism, however, remains elusive and future studies are needed. Computational analysis suggest that the dipole moment and geometry of molecule might be a good descriptors for the (Q)SAR studies of novel (thio)semicarbazides as potent antiserotonergic and antinociceptive agents.
